# Advances in the Understanding of the Transfer of Saccharides through NF Membranes in the Presence of Electrolytes by Coupling Quantum Mechanics and Thermodynamic Methods

**DOI:** 10.3390/membranes11050341

**Published:** 2021-05-05

**Authors:** Johanne Teychené, Hélène Roux-de Balmann, Sylvain Galier

**Affiliations:** 1 Toulouse Biotechnology Institute (TBI), Université de Toulouse, CNRS INRA, INSA, CEDEX 04, F-31077 Toulouse, France; rumpala@insa-toulouse.fr; 2 Laboratoire de Génie Chimique, Université de Toulouse, INPT, UPS, CEDEX 09, F-31062 Toulouse, France; roux@chimie.ups-tlse.fr; 3 CNRS, Laboratoire de Génie Chimique, CEDEX 09, F-31062 Toulouse, France

**Keywords:** nanofiltration, mass transfer modelling, saccharide, electrolyte, hydration number, interactions, ion coordination number

## Abstract

Different studies have shown that the presence of electrolytes modifies the nanofiltration performances and that the variation of the neutral solute transfer is mainly governed by the modification of the solute properties. The objective of this work is to strengthen the understanding of the impact of the ion composition and to progress in the long-term objective for the prediction of the nanofiltration performances. The methodology is based on the comparison of the hydration properties obtained by experimental and theoretical approaches with the mass transfer of saccharides. The key role of the saccharide hydration number to understand the impact of the ionic composition on the saccharide transfer is clearly demonstrated. Moreover, it is established that the number of saccharide/cation interactions, which increases with the cation coordination number, is a key parameter to understand the mechanisms governing the impact of the nature of the cation on the saccharide mass transfer modification. Finally, correlations are obtained between the saccharide hydration number decrease and the variation of the saccharide radius calculated using a hydrodynamic model for different ionic compositions and operating modes (diffusion and filtration). From these results, it could be possible to evaluate the saccharide transfer for a given saccharide/electrolyte system transfer.

## 1. Introduction

Membrane separation techniques, such as nanofiltration (NF), constitute an interesting alternative for developing high-performance processes capable of treating fluids containing variable proportions of organic and mineral solutes [1,2]. However, NF development is still hampered, in particular due to the difficulty of predicting its performances when the ionic composition of the fluids to be treated varies or when new, more demanding uses are considered [3,4,5,6].

In recent years, various studies have shown that the presence of electrolytes significantly modifies the performances of NF due to the variation of the neutral solutes transfer [7,8,9,10,11,12,13]. For instance, Wang et al. [14] showed that glucose transfer increases in the presence of NaCl, and that this increase is all the more important as the NaCl concentration is high. Neutral solute transfer in nanofiltration is essentially governed by steric effects, depending on the ratio between the size of the solutes and the size of the membrane pores. Thus, to explain the increase in solute transfer in the presence of electrolytes, several assumptions have been made [15,16], such as an increase in apparent pore size [11,12,14,17], a different pore size distribution in the membrane [12], an increase in the solution viscosity [18], a change in the interactions between the solute and the membrane [15] and finally the partial dehydration of the solute [7,8,9,10,11,17,19]. 

The relation between the solute hydration properties and the dehydration phenomenon has also been proposed to explain the impact of the nature of the ions (valence and size) on their transfer through membranes [20]. For instance, Zhao and al. [21,22] observed a good agreement between the decrease in the ion hydrated radius and the increase in the membrane ion exchange capacity. Moreover, these authors also pointed out that the ions need to eliminate a part of their coordinated water molecule to transfer through the membrane. The ion dehydration sequence has been correlated with ion membrane selectivity and explained by the ion hydration energy which reflects the ion/water interactions [21].

Concerning the impact of the presence of ions on the neutral solute transfer, the solute dehydration has been confirmed within the works of Bouranene and et al. [7,19] and Boy and et al. [9,10]. More precisely, in the case of the investigation of the transfer of saccharides in the presence of electrolytes, it has been pointed out that the impact of the modification of the membrane properties on saccharide transfer is negligible and that the transfer is, therefore, essentially governed by the modification of the solute properties. The assumption of saccharide dehydration has been validated from the good correlations obtained between the variation of the saccharide flux, determined in both diffusion and filtration operating modes for a given electrolyte, and the variation of the saccharide apparent molar volume, which reflects the saccharide hydration state [9,10]. Moreover, it has been demonstrated that these results are physically relevant to the dehydration phenomenon since the solute radius variation in the presence of electrolytes, calculated from the mass transfer modelling, using a hydrodynamic model, was found to correspond to a fraction of a water molecule [10]. These works also clearly demonstrated the impact of the ion valence on the increase in the saccharide transfer (divalent > monovalent). More precisely, it has been shown that the increase in saccharide transfer, due to the impact of the electrolyte on the saccharide properties, is all the more important as the ions in solution are hydrated (divalent ions > monovalent ions). However, concerning the impact of the electrolyte nature, it has not been possible to establish a clear quantitative relationship between the mass transfer modification and the variation of the saccharide apparent molar volume [9]. 

Then, in order to further understand the impact of the ionic composition on the transfer of a neutral solute, recent investigations using complementary approaches at different scales were carried out to investigate the mechanisms governing the saccharide/electrolyte interactions.

First, at the macroscopic scale, a thermodynamic method, based on the determination of the apparent molar volume of saccharides in the presence of electrolytes, was used to characterize the saccharide hydration. The determination of the volumetric properties of saccharides (xylose, glucose and sucrose) in different electrolytes (LiCl, NaCl, KCl, Na_2_SO_4_, K_2_SO_4_, CaCl_2_, MgCl_2_ and MgSO_4_) confirmed that most of the contributions to saccharide dehydration in the presence of electrolytes is due to the ion valence (divalent ions > monovalent ions). Additionally, it was also observed that the saccharide dehydration increases according to the following sequences: Na^+^ < K^+^ < Mg^2+^ < Ca^2+^ and Cl^−^< SO_4_^2−^, and that the anions (Cl^−^ or SO_4_^2−^) have no influence on the cation sequence [23]. Moreover, the saccharide hydration numbers, which reflect the solute hydration state, were also determined for the various electrolytes and the different concentrations investigated from the corresponding partial molar volume of saccharides [24]. 

More recently, the interactions in saccharide/electrolyte systems were also determined at the microscopic scale using a theoretical method (DFT, theory of density functionality). First, the computation of the ion hydration properties in water confirmed that the presence of an anion (Cl^−^ or SO_4_^2−^) does not lead to a change in the first cation hydration layer (Na^+^, K^+^, Mg^2+^ and Ca^2+^) [25]. Considering this last point, the saccharide/electrolyte interactions have been characterized in saccharide/cation/water systems showing that in such systems, cations and saccharides are both dehydrated [26]. In agreement with the results obtained from the volumetric properties, quantum mechanics calculations have shown that the saccharide dehydration increases with the ion valence and that the saccharide dehydration increases according to the following sequences: Na^+^ < K^+^ and Mg^2+^ < Ca^2+^. Simulation results showed that the number of saccharide/cation interactions increases with the cation coordination number in water (i.e., the number of water molecules directly interacting with the cation), and that this increase is directly linked to an increase in the saccharide dehydration. 

Finally, ion and saccharide properties, obtained from thermodynamic data as well as simulation works, were compared in order to rationalize the relation between the saccharide hydration number and the hydration properties of ions [24]. It was shown that the saccharide hydration number decreases linearly with the cation coordination number, regardless of the cation valence. Divalent cations have a greater impact than monovalent ones due to the stronger electrostatic interactions with the saccharide hydroxyl groups which induce higher coordination numbers. For a given cation valence, the coordination number increases with the size of the cation. This allows the cation to create stronger interactions with the saccharide and then to dehydrate the saccharide. Thus, the combination of the characterization of the hydration properties of both ions and saccharides at different scales has shown that the knowledge of the hydration properties of ions makes it possible to evaluate the saccharide hydration number. 

As previously mentioned, mass transfer models, such as the hydrodynamic model, can be considered to investigate the impact of the dehydration phenomenon on the mass transport of neutral solutes in confined space through the determination of the solute radius [10]. However, it is still difficult to integrate the influence of the confined space and, more precisely, the impact of the solution/membrane interactions on the mass transfer of molecules. Interactions at the membrane interface are related to space as well as time scales. Thus, knowledge at the molecular scale of the behaviors of aqueous solutions, containing organic and inorganic species, which interact with the membrane surfaces, in confined spaces, are essential to their understanding. In molecular simulation, given the size of the macromolecular systems to study, most of the models used to simulate systems containing membrane and aqueous solution are on a “coarse grain” scale due to their high computational effort (time and space). For instance, molecular dynamics simulation has recently been used to model the mass transfer through NF/RO membranes [27], showing interesting results in the case of single ions or water, although it is impossible to compute “practical” conditions in terms of pressure, for instance. Moreover, in any case, it is impossible to implement non-covalent interactions in molecular dynamics, which is the key point for the investigation of the dehydration phenomenon.

Then, based on these recent results, the aim of this paper is to advance the knowledge of the mechanisms and the estimation of the mass transfer of neutral solutes through nanofiltration membranes as a function of the ionic composition. More precisely, the objective of this work is to strengthen the understanding of the impact of the ion nature, especially for a given valence, since it has not been completely explained to date. The envisaged methodology is based on the comparison of the hydration properties obtained by experimental and theoretical approaches (volumetric properties from a thermodynamic method and quantum mechanics data) with the mass transfer of saccharides through an NF membrane. Thus, correlations between the solute hydration properties obtained at different scales [23,24,25,26] and the mass transfer parameters, which characterize the impact of the electrolyte on the saccharide transfer, are investigated to improve the understanding of the impact of the nature (cation, anion, size, valence, etc.) and the concentration of the ions. Special attention is paid to the saccharide hydration number and to the coordination number of ions since it has been shown that these hydration properties are closely linked and relevant to describe and understand saccharide dehydration in the presence of electrolytes. Then, in order to progress in the long-term objective for the prediction of the NF performances, the relation between the variation of the saccharide hydration number in the presence of the electrolyte and the modification of the saccharide radius calculated using a hydrodynamic model is investigated.

## 2. Materials and Methods

### 2.1. Mass Transfer Experiments

In order to understand and highlight the impact of the nature of the ion on the modification of saccharide transfer in nanofiltration, additional transfer measurements were performed in this work. The experiments were performed in the diffusion operating mode (concentration gradient driving force) to avoid the impact of ion retention on saccharide mass transfer [9]. 

**Figure 1:**
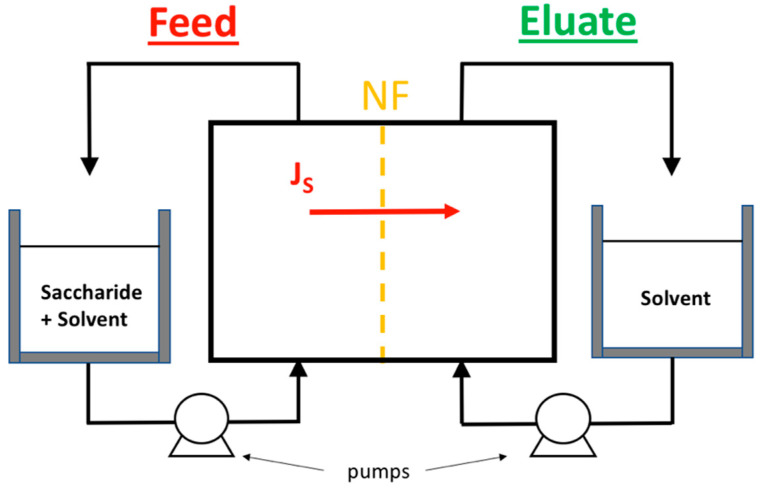
Nanofiltration experimental device (solvent = water for saccharide/water systems; solvent = electrolyte for saccharide/electrolyte systems).

The mass transfer study was carried out for glucose (180.16 g mol^−1^) in electrolytes containing Cl^−^ (NaCl, KCl, MgCl_2_ and CaCl_2_) with a purity > 99%, for given glucose and cation concentrations (1 mol·kg^−1^). The schematic device of the experimental setup is shown in Figure 1. The mass transfer measurements were conducted using the same nanofiltration membrane (Dow Filmtec NF membrane, polyamide active layer), diffusion cell and experimental procedure as described in Boy and et al. [9].

The diffusion experiments were carried out with glucose/water and glucose/electrolyte solutions at 25 °C after the membrane soaking with the given electrolyte for 24 h. 

For any set of experiments, the glucose concentration was measured by HPLC with a Jasco system, using a Shodex column (SH 1011) and a Jasco RI 2031 refractometer. The mobile phase was a 0.02 mol·L^−1^ H_2_SO_4_ solution at 0.8 mL·min^−1^. The injection volume was 20 µL, and the column temperature as 50 °C.

The experiments were carried out at least twice. For all experiments, the evolution of the number of moles of glucose as a function of time was linear, confirming that the glucose concentration gradient was constant throughout the experiment. The same membrane sample was used for all the experiments.

### 2.2. Mass Transfer Parameters

The specific protocol developed by Boy et al. [9] was used to dissociate and quantify the contribution of the modification of the solutes or the membrane properties to the transfer of saccharides. The impact of electrolytes on the membrane properties was characterized by the saccharide flux measured in water, *J_S,W_*. The impact of electrolytes on the saccharide properties was estimated from the additional saccharide flux, ∆*J*, expressed as the difference between the saccharide flux measured in saccharide/electrolyte systems, *J_S,El_*, and the one determined in saccharide/water systems:

(1) $$\Delta J=J_{S,El}-J_{S,W}.$$

In diffusion mode, the solute transfer was modelled using a hydrodynamic model [28] in which the solute flux is related to the driving force (concentration gradient) through the characteristic parameters of the systems studied [10]:

(2) $$J_{S}=\frac{\phi \times K_{d}\times D_{\infty }\times A_{k}}{L}\;\Delta C,$$

where *ϕ* is the partitioning coefficient; *K_d_* is the hindrance factor for diffusion; *D*_∞_ (m^2^·s^−1^) is the diffusion coefficient of the solute at infinite dilution; *A_k_* is the membrane porosity; *L* (m) is the pore length; and Δ*C* (mol·m^−3^) is the concentration difference between the feed and eluate compartments, i.e., across the membrane. The partition coefficient, *ϕ*, and the hindrance factor for diffusion, *K_d_*, are related to the pore radius, *r_P_*, and the solute radius, *r_S_*. The equations are given in ref. [10].

Then, the solute radius in the electrolyte, *r_S,El_*, can be obtained by assuming that the variation of the saccharide transfer in the presence of electrolytes compared to that in water is due to a decrease in the solute radius (*r_P_* is assumed constant and independent of the electrolyte composition). More precisely, the solute radius in the electrolyte was determined from the comparison between the ratio of the experimental and calculated values of the diffusion flux in the electrolyte, *J_S,El_*, and in water, *J_S,W_*, using a mean square method.

Then, the modification of the solute radius in the presence of electrolytes, Δ*r_S_*, was calculated according to the following expression:

(3) $$\Delta r_{S}=r_{S,El}-r_{S,W},$$

where *r_S,W_* is the saccharide radius in water.

The values of the membrane pore radius considered in this work for xylose, glucose and sucrose are 0.45, 0.50 and 0.54 nm, respectively. They were obtained for the same membrane with saccharide water systems in filtration operating mode (pressure driven driving force) [10,29]. Indeed, it is commonly reported that increasing values are obtained for increasing solute size due to the pore size distribution, since strongly retained solutes are transferred through the larger pores [12,29].

## 3. Results

The objective is to improve the understanding and evaluation of mass transfer in nanofiltration in the presence of electrolytes. In order to progress in this objective, the methodology developed in the present work is based on the comparison between quantities characterizing the impact of the ionic composition on the saccharide transfer and the hydration properties of solutes. These properties are obtained by different methods characterizing the hydration at macroscopic and microscopic scales.

### 3.1. Mass Transfer and Saccharide Hydration Number

#### 3.1.1. Influence of the Cation

The results obtained in this work with glucose/water and glucose/electrolyte systems in diffusion mode and in the presence of various electrolytes containing Cl^−^ with different cations, are given in Table 1.

Values obtained in the present study are of the same order of magnitude than those obtained in a previous work [9]. The differences can be explained by the use of different membrane samples (derived from the same commercial reference). Such differences have already been observed.

The saccharide fluxes in water, *J_S,W_*, measured for the membrane conditioned with NaCl, MgCl_2_ and CaCl_2_ were identical. For the membrane soaked with KCl, the values of *J_S,W_* were lower than those measured in the other electrolytes.

Results showed that the saccharide flux increased in the presence of electrolytes, *J_S,El_* > *J_S,W_* (factor of 1.5 to 6). The glucose flux in water ranged between 2.7 and 4.7 × 10^−7^ mol·m^−2^·s^−1^, while it ranged between 8.6 and 25.2 × 10^−7^ mol·m^−2^·s^−1^ in glucose/electrolyte systems.

Results reported in Table 1 show that the additional flux of glucose, ∆*J*, into the electrolytes followed the given sequence: NaCl < KCl < MgCl_2_ < CaCl_2_. Although for the membrane soaked in KCl, *J_S,W_* was smaller than *J_S,W_* for the membrane soaked in NaCl, *J_S,El_* in the presence of KCl still remained larger than the glucose flux in NaCl. In spite of the singularity of the glucose flux in water for a membrane soaked with KCl, which is not investigated in this work, one can notice that there was no singularity of K^+^ on the glucose flux in the presence of electrolyte. Therefore, the additional glucose flux, ∆*J*, depends on the nature of the electrolyte, and the increase in saccharide flux is greater in electrolytes containing divalent cations than in electrolytes containing monovalent ones.

As specified in the introduction, the saccharide hydration number at an infinite dilution, *n_H_*, is more relevant than the variation of the partial molar volume to reveal the solute hydration degree. The *n_H_* glucose values, calculated from thermodynamic data, are reported in Table 1 [23]. The comparison of n_H_ values in different electrolyte showed that glucose dehydration increased according to the following sequence: Na^+^ < K^+^ < Mg^2+^ < Ca^2+^. Thus, with regard to the influence of the nature of the cation, a good agreement was obtained between the two sequences characterizing the increase in saccharide transfer and its dehydration in the presence of electrolyte.

**Figure 2:**
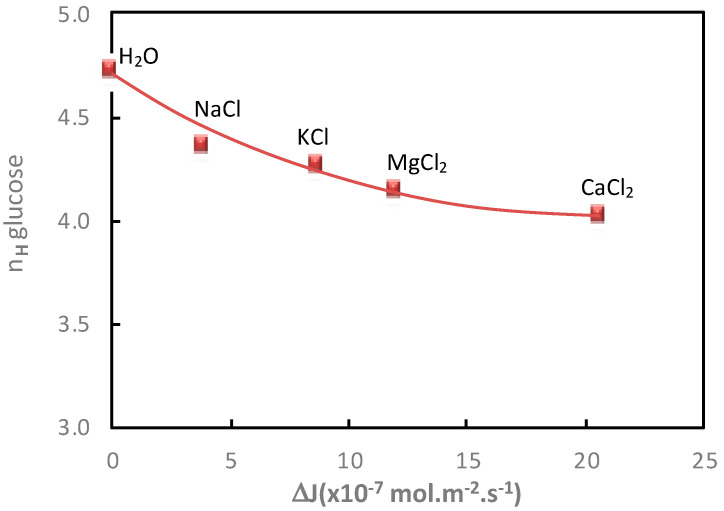
Impact of the cation nature. Correlation between the glucose hydration number, *n_H_*, and the glucose additional flux, Δ*J*, in the presence of different electrolytes (NaCl, KCl, MgCl_2_ and CaCl_2_). Cation and glucose molality = 1 mol·kg^−1^; diffusion mode; T = 25 °C. Glucose hydration number in water: *n_H_* = 4.72.

Then, in order to further understand the impact of the cation on the additional saccharide flux, the glucose hydration numbers and the glucose additional flux values measured in the presence of electrolytes containing Cl^−^ (NaCl, KCl, MgCl_2_ and CaCl_2_) were compared and represented in Figure 2.

It can be observed that a good relationship was obtained between the decrease in the glucose hydration number and the increase in glucose flux, under diffusion conditions, in the presence of electrolytes containing different cations. It is interesting to note that it was not possible to establish this kind of correlation from the variation of the apparent molar, which also revealed the solute hydration state [9].

#### 3.1.2. Influence of the Anion

In order to confirm the key role of the saccharide hydration number, the additional saccharide fluxes obtained for various ionic compositions and operating modes (diffusion and filtration) obtained in a previous study were also considered (Boy et al. [9]).

**Figure 3:**
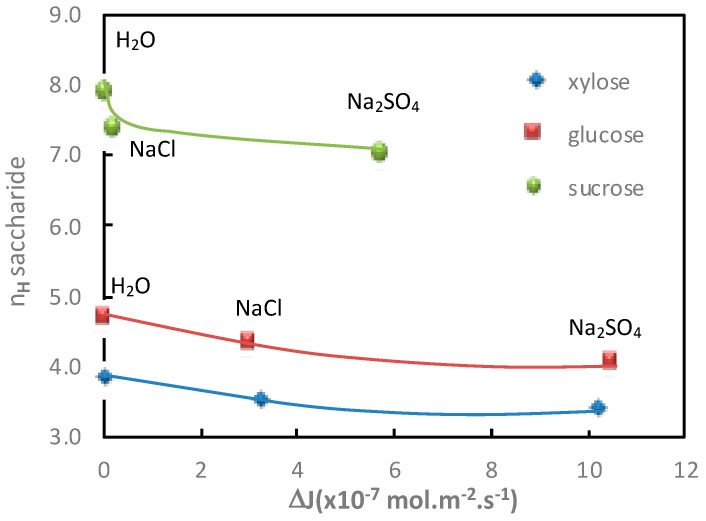
Impact of the anion nature. Correlation between the saccharide hydration number, *n_H_*, and the saccharide additional flux, Δ*J*, in the presence of different electrolytes (NaCl and Na_2_SO_4_). Cation and saccharide molality = 1 mol·kg^−1^; diffusion mode; T = 25 °C.

The saccharide additional flux values (xylose, glucose and sucrose) obtained in diffusion mode and in the presence of electrolytes containing Na^+^ with different anions (Cl^−^ and SO_4_^2−^) for a given cation molality (1 mol·kg^−1^), as well as the corresponding saccharide hydration number, are given in Table 2 and represented in Figure 3.

Figure 3 shows that the greater increase in saccharide flux with Na_2_SO_4_ than with NaCl is related to higher saccharide dehydration in the presence of Na_2_SO_4_. This confirms thermodynamic results [23], showing that saccharide dehydration increases according to the following sequence: Cl^−^ < SO_4_^2−^. Moreover, as observed with the cations, a good relationship was obtained between the saccharide hydration number and the increase in saccharide flux, under diffusion conditions, in the presence of electrolytes containing different anions.

#### 3.1.3. Influence of the Electrolyte Concentration

**Figure 4:**
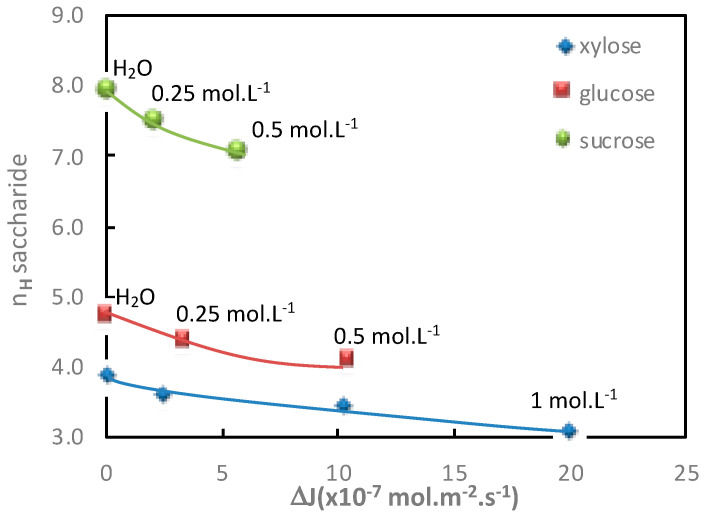
Impact of the electrolyte concentration. Correlation between the saccharide hydration number, *n_H_*, and the saccharide additional flux, Δ*J*, in the presence of Na_2_SO_4_. Saccharide = 1 mol·L^−1^; diffusion mode; T = 25 °C.

**Figure 5:**
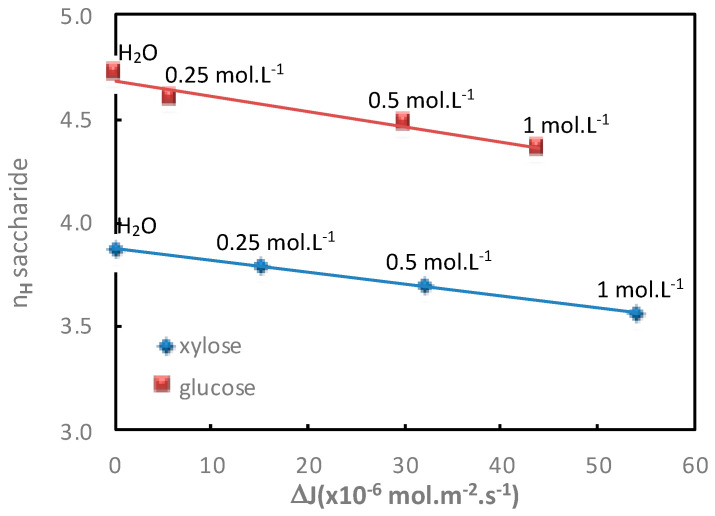
Impact of the electrolyte concentration. Correlation between the saccharide hydration number, *n_H_*, and the saccharide additional flux, Δ*J*, determined at a permeate flux of *J_v_* = 0.5 × 10^−5^ m·s^−1^, in the presence of NaCl. Saccharide = 0.1 mol·L^−1^; filtration mode; T = 25 °C.

The additional saccharide flux values obtained for various electrolyte concentrations in both diffusion mode (Na_2_SO_4_) and filtration mode (NaCl) with the corresponding saccharide hydration number are given in Table 3 and Table 4 and represented in Figure 4 and Figure 5, respectively.

In both operating modes (diffusion or filtration), Figure 4 and Figure 5 show a good relationship between the increase in additional saccharide flux and the corresponding saccharide hydration number decrease for increasing electrolyte concentrations.

To summarize, it was demonstrated that the saccharide hydration number is a key parameter to describe the impact of the presence of electrolytes on the saccharide mass transfer since good correlations were obtained for different ionic compositions (ion nature and concentration) and operating modes (diffusion and filtration).

### 3.2. Mass Transfer and Cation Hydration Properties

In our previous work, an original approach combining a quantum mechanics technique (DFT) and experimental measurements (thermodynamic properties) was developed to explain the relationship between the saccharide hydration and the hydration properties of cations [24]. More precisely, it was found that the saccharide hydration number, *n_H_*, decreases linearly with the increase in the cation coordination number, *CN*, which reflects the ion’s ability to form direct interactions with the saccharide hydroxyl groups.

In our previous works, the solute hydration properties (ions and saccharides) in pure water and in mixture were studied via a full geometry optimization using the DFT method [24,25,26]. First, the ion coordination number, *CN*, which is defined as the number of water molecules directly bonded to the central ion in its solvation first layer, was determined [25]. Then, the number of interactions between the cation and the oxygen, *n_inter S/C+_*, defined as the number of oxygen belonging to the saccharide in direct interaction with the ion, was calculated [26]. Results concerning the ion coordination number and the number of interactions between glucose and different cations are given in Table 5 [25,26]. In saccharide/cation/water systems, results showed that saccharides are all the more dehydrated as the number of saccharide/cation interactions increases, *n_inter S/C+_* [26]. The number of saccharide/cation interactions increases with the ion coordination number in water, *CN*.

For a given cation valence, the saccharide dehydration sequence, as a function of the cation, was found to be identical to that obtained with the thermodynamic method (Na^+^ < K^+^ < Mg^2+^ < Ca^2+^).

**Figure 6:**
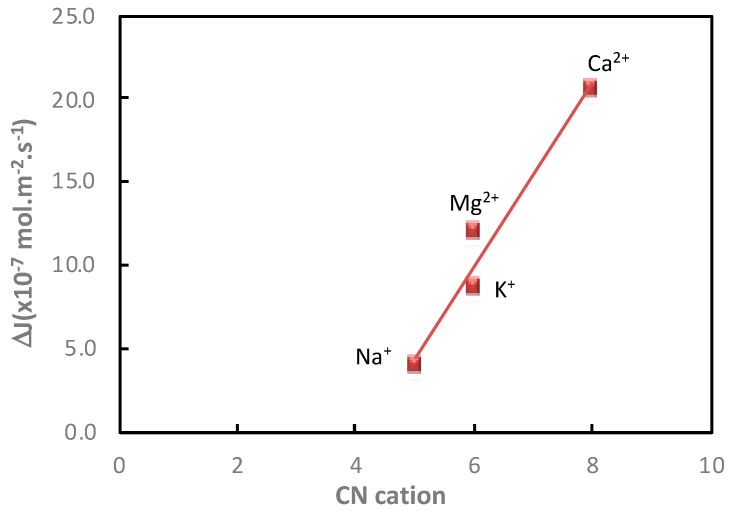
Impact of the cation. Evolution of the glucose additional flux, ∆*J*, measured in the presence of NaCl, KCl, MgCl_2_ and CaCl_2_ as a function of the cation coordination number, *CN*, of Na^+^, K^+^, Ca^2+^ and Mg^2+^. Cation and glucose molality = 1 mol·kg^−1^; diffusion mode; T = 25 °C.

In order to strengthen the understanding of the mechanisms governing the impact of the presence of electrolytes on the saccharide transfer, the additional saccharide flux and hydration properties of cations (*CN* cations) were compared (Figure 6).

It was observed that the additional glucose flux increased linearly with the cation coordination number of the electrolyte. However, it was not possible to distinguish the impact of the cations of different valences with the same coordination number (K^+^ and Mg^2+^) on the additional glucose flux. Contrarily, as observed in Table 5, the number of saccharide/cation interactions allowed us to distinguish K^+^ and Mg^2+^.

**Figure 7:**
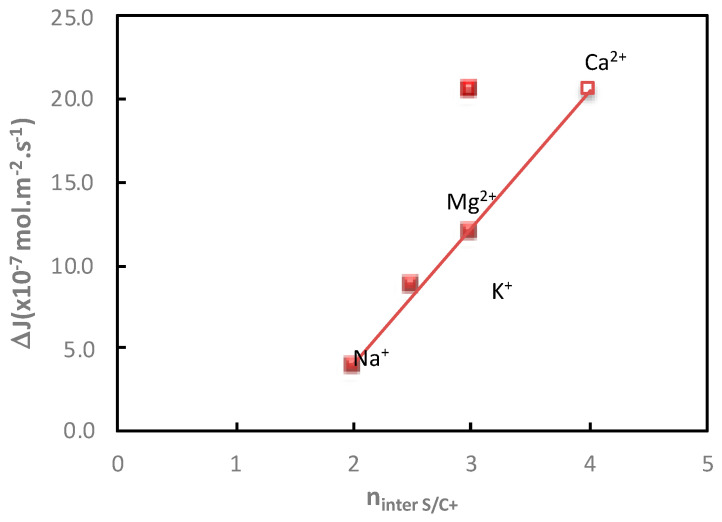
Impact of the cation. Evolution of the glucose additional flux, ∆*J*, in the presence of NaCl, KCl, MgCl_2_ and CaCl_2_ as a function of the number of glucose/cation interactions, *n_inter S/C+_*. Cation and glucose molality = 1 mol·kg^−1^; diffusion mode; T = 25 °C.

Figure 7 shows the evolution of the additional saccharide flux as a function of the number of saccharide/cation interactions. One can observe that the increase in glucose flux is related to the increase in the number of saccharide/cation interactions and thus to the increase in saccharide dehydration. A linear relationship was established between the number of saccharide/cation interactions and the additional glucose flux, except in the presence of Ca^2+^. Indeed, it was not possible to distinguish the impact of the cations which had the same number of saccharide/cation interactions (*n_inter_* Glucose/Mg^2+^ = *n_inter_* Glucose/Ca^2+^ = 3). The variation of the number of saccharide/cation interactions is very sensitive to small variations in saccharide/cation interactions. Thus, one can expect that the number of glucose/cation interactions should be higher in the presence of Ca^2+^ than in the presence of Mg^2+^, as previously observed with sucrose [26]. Indeed, Ca^2+^ is able to create more interactions with glucose than Mg^2+^, due to its higher coordination number. Thus, as observed in Figure 7, a linear relationship can be obtained between the number of glucose/cation interactions and the glucose additional flux, by increasing the number of glucose/Ca^2+^ interactions by one unit (*n_inter_* Glucose/Ca^2+^ = 4). This point merits further investigation.

Consequently, it was demonstrated that both the cation coordination number, *CN*, and the number of saccharide/cation interactions, *n_inter S/C+_*, are necessary to understand the mechanisms governing the impact of the nature of the cation on the saccharide mass transfer modification in the presence of electrolytes.

### 3.3. Hydration Number and Saccharide Radius

As mentioned in the introduction, the performances of the nanofiltration process depend on the combination of quite complex and imbricated mechanisms, such as the impact of the ion composition on the transfer of neutral species investigated in this work. As a result, it was barely possible to predict them, and this is a bottleneck for the development of this operation. This problem will be solved once the parameters required for the mass transfer modelling, such as membrane and solutions properties, are accessible. It is a long-term objective that requires the elucidation of the phenomena.

Then, based on the better understanding of the mechanisms governing the mass transfer of saccharide in the presence of electrolyte, i.e., the phenomena of saccharide dehydration, one can expect to progress in this long-term objective.

Typically, the mass transfer of a neutral solute can be described and predicted using a hydrodynamic model [10,28]. More precisely, the neutral solute transfer is fixed by the membrane properties (pore radius, membrane porosity and pore length), the solute radius and the operating conditions. For instance, the saccharide transfer in the presence of electrolytes was modelled by Boy et al. to evaluate the saccharide radius in the presence of electrolytes, *r_S,El_*, and to deduce the variation of the saccharide radius, Δ*r_S_* (see Equation (3)), in both diffusion and filtration operating modes [10].

In the present work, the correlation between the saccharide flux variation and the saccharide hydration number for various ionic compositions was clearly demonstrated. Then, in order to progress in the mass transfer prediction, the relation between the saccharide hydration number in the presence of the electrolyte and the variation of the saccharide radius calculated from the modelling of the saccharide flux using the hydrodynamic model, was investigated.

The membrane properties and, more precisely, the membrane pore radius, *r_p_*, was assumed to be constant since it was shown that the saccharide fluxes measured in water, *J_S,W_*, are weakly influenced by the nature of the electrolyte used for the membrane soaking. Usually, the membrane pore radius is obtained in filtration operating mode from the modelling of a neutral solute transfer using the hydrodynamic model.

In diffusion mode, the saccharide radius in the presence of electrolyte, *r_S,El_*, and the variation of the saccharide radius, Δ*r_S_*, were determined according to the procedure developed by Boy et al. [10] and described in Section 2.2. The values obtained with various cations, anions and electrolyte concentrations are given in Table 1, Table 2 and Table 3, respectively. The saccharide radius in the presence of electrolytes, *r_S,El_*, and the variation of the saccharide radius, Δ*r_S_*, in filtration mode were obtained from [10] and given in Table 4.

**Figure 8:**
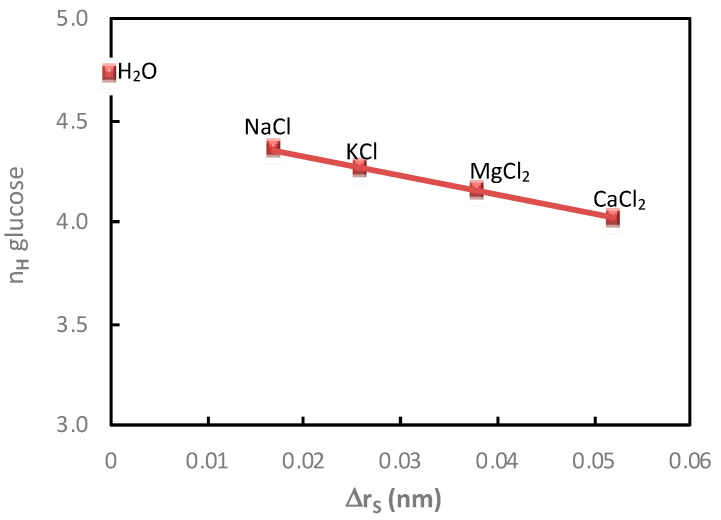
Impact of the cation. Correlation between the glucose hydration number, *n_H_*, and the variation of the glucose radius, Δ*r_S_*, in the presence of different electrolytes (NaCl, KCl, MgCl_2_ and CaCl_2_). Cation and glucose molality = 1 mol·kg^−1^; diffusion mode; T = 25 °C. Glucose hydration number in water: *n_H_* = 4.72.

**Figure 9:**
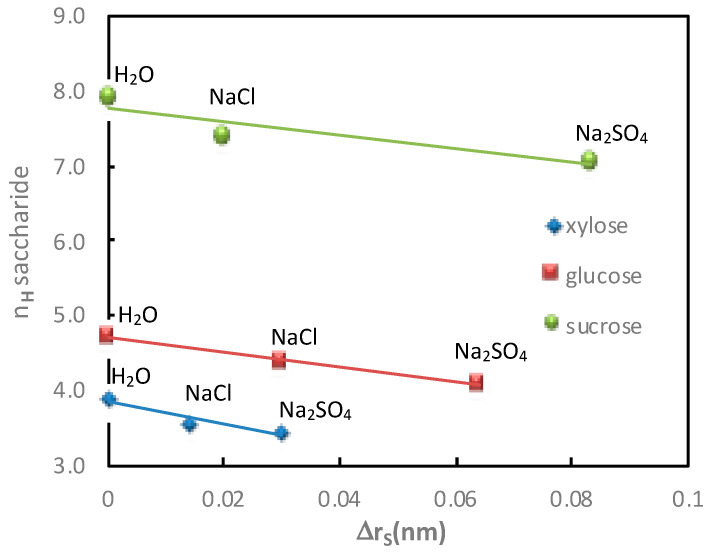
Impact of the anion. Correlation between the saccharide hydration number, *n_H_*, and the variation of the saccharide radius, Δ*r_S_*, in the presence of different electrolytes (NaCl and Na_2_SO_4_). Cation and saccharide molality = 1 mol·kg^−1^; diffusion mode; T = 25 °C.

The variations of saccharide hydration numbers as a function of the saccharide radius variation, calculated in diffusion mode, as a function of the cation and anion nature, are reported in Figure 8 and Figure 9, respectively.

As expected, it was observed that the variation of the saccharide radius in the presence of electrolytes increased, i.e., lower saccharide radius, with the decrease in the saccharide hydration number. More precisely, it is worth noting that in the condition investigated in this work, a linear relation was observed in the presence of electrolytes containing a given anion (various cations; Figure 8) or a given cation (various anions; Figure 9). The slight deviation observed for the glucose hydration number in water merits further investigation.

**Figure 10:**
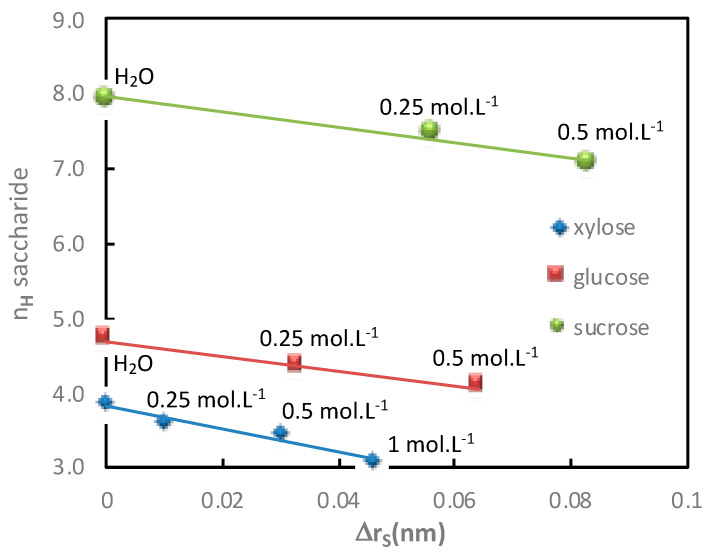
Impact of the electrolyte concentration. Correlation between the saccharide hydration number, *n_H_*, and the variation of the saccharide radius, Δ*r_S_*, in the presence of Na_2_SO_4_ (various concentrations indicated in the figure). Saccharide = 1 mol·L^−1^; diffusion mode; T = 25 °C.

**Figure 11:**
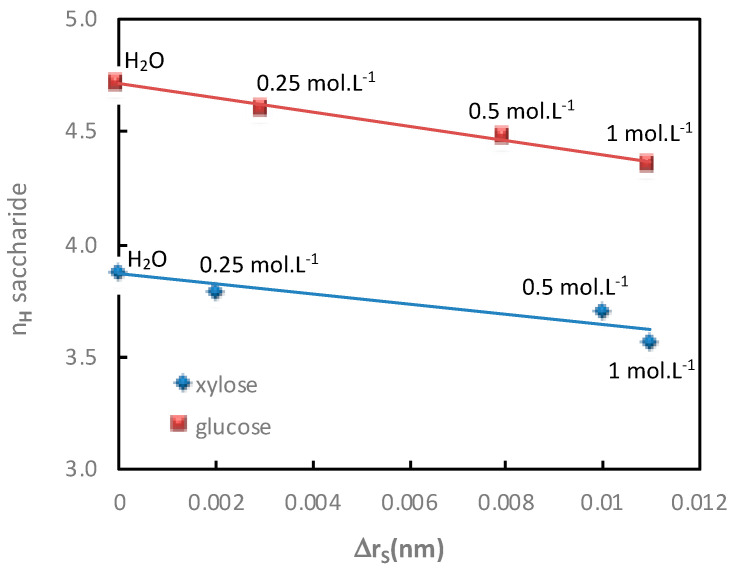
Impact of the electrolyte concentration. Correlation between the saccharide hydration number, *n_H_*, and the variation of the saccharide radius, Δ*r_S_*, in the presence in the presence of NaCl (various concentrations indicated in the figure). Saccharide = 0.1 mol·L^−1^; filtration mode; T = 25 °C.

The variations of saccharide hydration numbers as a function of the saccharide radius variation, calculated in both diffusion and filtration operating modes, as a function of the electrolyte concentration, are reported in Figure 10 and Figure 11, respectively.

In both operating modes (diffusion or filtration), it was shown again that the variation of the saccharide radius in the presence of electrolytes of various concentrations increased linearly with the decrease in the saccharide hydration number.

Consequently, the key role of the saccharide hydration number was strengthened, since clear correlations with the saccharide radius in the presence of electrolytes were obtained for different ionic compositions (ion natures and concentrations) and operating modes (diffusion and filtration). Moreover, linear correlations were obtained for the conditions considered in this work.

As previously discussed, the saccharide hydration number in the presence of electrolytes can be determined from the experimental determination of thermodynamic properties (volumetric properties) [23] or evaluated from the correlations with the ion coordination number (see [24]). Thus, using the previous linear correlations, it is possible to evaluate the variation of the saccharide radius according to the ionic composition and to estimate the saccharide radius in the presence of electrolytes. Finally, for a given saccharide/electrolyte system, knowing the saccharide radius in water as well as the membrane pore radius, it could be possible to predict the saccharide transfer using the hydrodynamic model.

## 4. Conclusions

Various studies focusing on the study of the influence of ionic composition on the transfer of neutral solutes across nanofiltration membranes have shown that the increase in the transfer of the neutral solute in the presence of electrolytes is mainly governed by the modification of the neutral solute hydration properties due to solute/electrolyte interactions. Recently, investigations on the mechanisms governing the saccharide/electrolyte interactions using complementary approaches at different scales have shown that the saccharide hydration numbers in the presence of electrolytes are closely linked to the hydration properties of the ions. Thus, the objectives of the present work were to strengthen the understanding of the impact of the ion nature, especially for a given valence, since it has not been completely explained to date, and to progress towards the long-term objective in the prediction of nanofiltration performances.

The influence of the nature of the cation on the transfer of glucose in diffusion mode was first investigated. The glucose hydration numbers, calculated from thermodynamic data, and the glucose additional flux values measured through an NF membrane in the presence of electrolytes containing Cl^−^ (NaCl, KCl, MgCl_2_ and CaCl_2_), were compared. A good agreement was obtained between the increase in the glucose transfer and its hydration number in the presence of electrolytes according to the following sequence: Na^+^ < K^+^ < Mg^2+^ < Ca^2+^. This result was confirmed from the additional saccharide flux values obtained for various ionic compositions and operating modes (diffusion and filtration). Consequently, the key role of the saccharide hydration number to understand the impact of the ionic composition on the saccharide transfer through an NF membrane was clearly demonstrated.

Second, in order to strengthen the understanding of the mechanisms governing the impact of the presence of electrolytes on the saccharide transfer, the additional saccharide flux and hydration properties of cations were compared. Indeed, on one hand, the relation between the saccharide hydration number and the cation coordination number has been established in previous works, and on the other hand, the correlation between the saccharide hydration number and the saccharide mass transfer was demonstrated in the present work. Thus, it was demonstrated that the number of saccharide/cation interactions, n_inter S/C+_, which increases with the cation coordination number, is a key parameter to understand the mechanisms governing the impact of the nature of the cation on the saccharide mass transfer modification in the presence of electrolyte.

Finally, in order to progress in the long-term objective toward the prediction of NF performances, the relation between the saccharide hydration number in the presence of the electrolyte and the variation of the saccharide radius calculated using the hydrodynamic model was investigated. The key role of the saccharide hydration number was strengthened since clear correlations with the saccharide radius in the presence of electrolyte were obtained for different ionic compositions and operating modes. Thus, based on the knowledge of the saccharide hydration number and the linear correlations established in this work, it could be possible to evaluate the saccharide transfer for a given saccharide/electrolyte system transfer using the hydrodynamic model. This last point merits further investigation.

## Figures and Tables

**Table 1:** Impact of the cation nature. Glucose flux in water, *J_S,W_*, and in the electrolyte, *J_S,El_*; additional glucose flux, ∆*J*; glucose hydration number, *n_H_* (from [24]); fitted glucose radius, *r_S,El_*, and its variation in the presence of electrolyte, Δ*r_S_*. Cation and glucose molality = 1 mol·kg^−1^; diffusion mode; T = 25 °C. Glucose hydration number and radius in water: *n_H_* = 4.72; *r_S,W_* = 0.363 nm.

Electrolyte	*J_S,W_*	*J_S,El_*	Δ*J*	*n_H_*	*r_S,El_*	Δ*r_S_*
	(×10^−7^ mol·m^−2^·s^−1^)	(×10^−7^ mol·m^−2^·s^−1^)	(×10^−7^ mol·m^−2^·s^−1^)		(nm)	(nm)
**NaCl**	4.7	8.6	3.9	4.36	0.346	0.017
**KCl**	2.7	11.4	8.7	4.26	0.337	0.026
**MgCl_2_**	4.6	16.6	12.0	4.15	0.325	0.038
**CaCl_2_**	4.6	25.2	20.6	4.02	0.311	0.052

**Table 2:** Impact of the anion nature. Additional saccharide flux (from [9]), Δ*J*; saccharide hydration number, *n_H_* (from [24]); fitted saccharide radius, *r_S,El_*, and its variation in the presence of electrolyte, Δ*r_S_*. Cation and saccharide molality = 1 mol·kg^−1^; diffusion mode; T = 25 °C.

Saccharide	Electrolyte	Δ*J*	*n_H_*	*r_S_*	Δ*r_S_*
		(×10^−7^ mol·m^−2^·s^−1^)		(nm)	(nm)
**Xylose**	water	0	3.88	0.319	0
	NaCl	3.2	3.57	0.305	0.014
	Na_2_SO_4_	10.2	3.45	0.289	0.030
**Glucose**	water	0	4.72	0.363	0
	NaCl	3.0	4.36	0.333	0.030
	Na_2_SO_4_	10.5	4.08	0.299	0.064
**Sucrose**	water	0	7.93	0.449	0
	NaCl	0.2	7.41	0.429	0.020
	Na_2_SO_4_	5.7	7.06	0.366	0.083

**Table 3:** Impact of the electrolyte concentration. Additional saccharide flux (from [9]), ∆*J*; saccharide hydration number, *n_H_* (from [24]); fitted saccharide radius, *r_S,El_*, and its variation in the presence of Na_2_SO_4_, Δ*r_S_*. Saccharide = 1 mol·L^−1^; diffusion mode; T = 25 °C.

Saccharide	[Na_2_SO_4_]	∆*J*	*n_H_*	*r_S_*	Δ*r_S_*
	(mol·L^−1^)	(×10^−7^ mol·m^−2^·s^−1^)		(nm)	(nm)
**Xylose**	0	0	3.88	0.319	0
	0.25	2.4	3.60	0.309	0.010
	0.5	10.2	3.45	0.289	0.030
	1	19.9	3.08	0.273	0.046
**Glucose**	0	0	4.72	0.363	0
	0.25	3.4	4.35	0.333	0.033
	0.5	10.5	4.08	0.299	0.064
**Sucrose**	0	0	7.93	0.449	0
	0.25	2	7.50	0.393	0.056
	0.5	5.7	7.06	0.366	0.083

**Table 4:** Impact of the electrolyte concentration. Additional saccharide flux (from [9]), ∆*J*, determined at a permeate flux of J_v_ = 0.5 × 10^−5^ m·s^−1^; saccharide hydration number, *n_H_* (from [24]); fitted saccharide radius, *r_S,El_*, and its variation in the presence of NaCl, Δ*r_S_* (from [9]). Saccharide = 0.1 mol·L^−1^; filtration mode; T = 25 °C.

Saccharide	[NaCl]	∆*J*	*n_H_*	*r_S_*	Δ*r_S_*
	(mol·L^−1^)	(x10^−7^ mol·m^−2^·s^−1^)		(nm)	(nm)
**Xylose**	0	0	3.88	0.319	0
	0.25	15	3.80	0.317	0.002
	0.5	32	3.71	0.309	0.010
	1	54	3.57	0.308	0.011
**Glucose**	0	0	4.72	0.363	0
	0.25	6	4.60	0.360	0.003
	0.5	30	4.48	0.355	0.008
	1	44	4.36	0.352	0.011

**Table 5:** Cation coordination number, *CN*, in pure water and interaction number between the cation and glucose, *n_inter S/C+_*, at 25 °C [25].

		Na^+^	K^+^	Mg^2+^	Ca^2+^
**Pure water**	*CN*	5	6	6	8
**Glucose**	*n_inter S/C+_*	2	2.5	3	3

## Data Availability

The data presented in this study are available on request from the corresponding author.
